# Self-supervised transformers predict dynamics of object-based attention in humans

**Published:** 2023-08

**Authors:** Hossein Adeli, Seoyoung Ahn, Nikolaus Kriegeskorte, Gregory J. Zelinsky

**Affiliations:** 1 Department of Psychology, Stony Brook University, New York, USA; 2 Department of Computer Science, Stony Brook University, New York, USA; 3 Zuckerman Mind Brain Behavior Institute, Columbia University, New York, USA

## Abstract

Spread of attention within objects has been proposed as a mechanism for how humans group features to segment objects. However, such a mechanism has not yet been implemented and tested in naturalistic images. Here, we leverage the feature maps from self-supervised vision transformers and propose a model of human object-based attention spreading and segmentation. The attention spreads within an object through the affinity signal between different patches of the image. We show that this model predicts reaction time patterns of people grouping objects in natural images by judging whether two dots are on the same object or on two different objects.

## Background

A fundamental problem that our visual system must solve is how to group parts of the visual input together into coherent whole objects (Peters & Kriegeskorte, 2021). The role of attention in solving this problem has been experimentally studied for decades (Treisman, 1996; Adeli, Ahn, & Zelinsky, 2022). A proposed solution is that attention can bind object features through activation spreading within an object using lateral connectivity in retinotopic visual areas (Roelfsema, 2023). However, the modeling work in this domain has focused on bottom-up, Gestalt cues, and clear object boundaries for how attention can spread within an object to bind its features (e.g. the ”growth cone” model (Jeurissen, Self, & Roelfsema, 2016)). A compelling model of primate vision, however, should be able to handle natural images where object boundaries are frequently ambiguous and bottom-up cues must be combined with prior object-specific knowledge.

**Figure 1:**
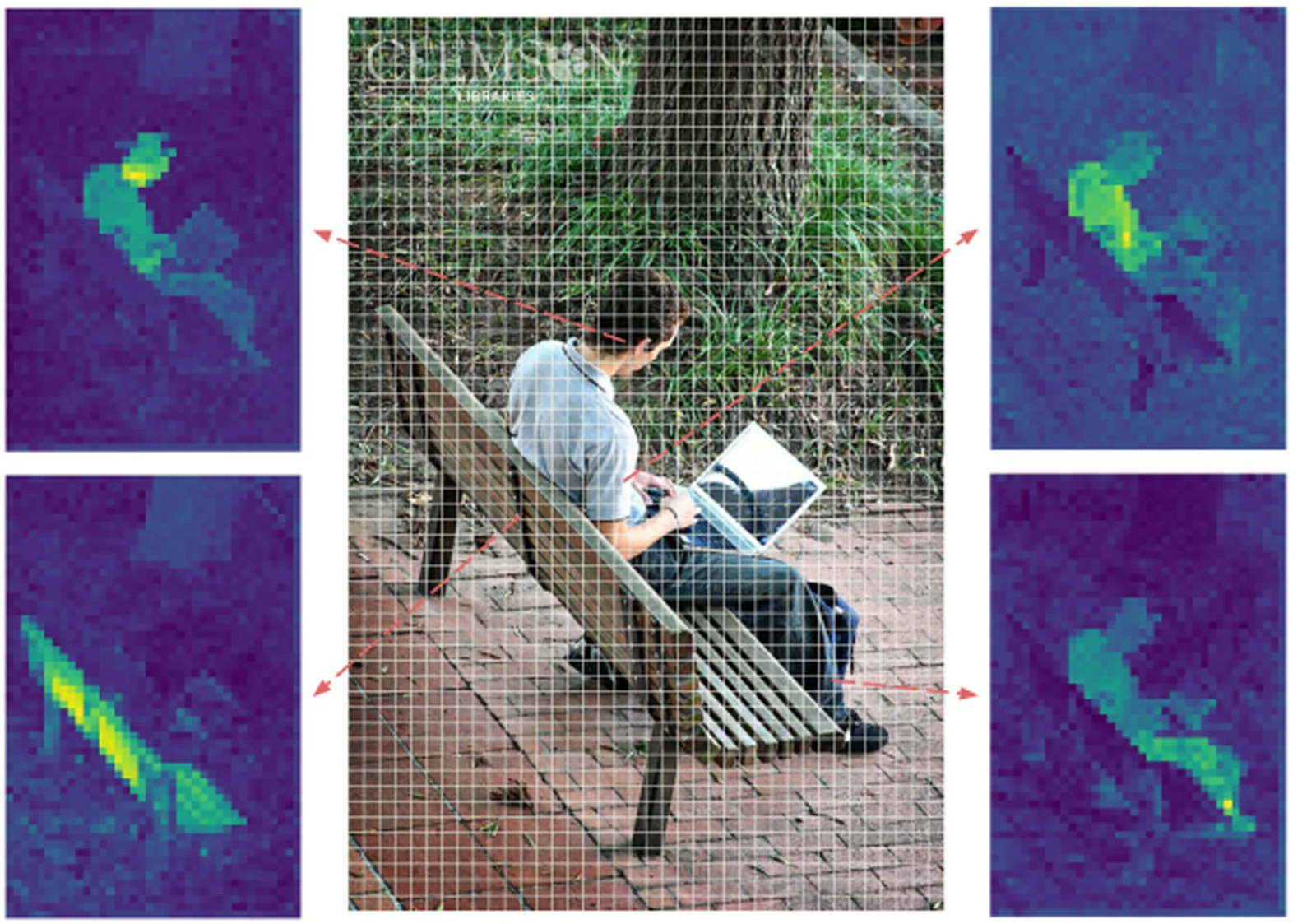
The affinity maps for select patches on the grid.

Building a model of object-based attention applicable to natural images requires the modeling of lateral connectivity between image regions that can guide the spread of attention. Recent vision transformers capture this connectivity and are therefore well-suited to address the spreading of object-based attention. In these models, the visual input is first divided into different patches (Fig. 1 center) that are then encoded as feature vectors called tokens (Dosovitskiy et al., 2020). At each layer of processing, a given token representing an image patch can update its value by interacting with and mixing (”attending” to) the values of all other tokens that it finds relevant. The selective nature of this mixing has motivated naming this process ”attention” in transformers (Vaswani et al., 2017). Recent work has shown that feature similarity between tokens, which we refer to as ”affinity” (Chen et al., 2022), in vision transformers trained with a self-supervised objective (e.g., distillation (Caron et al., 2021) or masked autoencoding (He et al., 2022)) begins to represent object-centric information (Wang et al., 2022), meaning the patches that have the highest affinity to a given patch are likely to be on the same object (Fig. 1). These dynamic pairwise interactions may serve a role similar to that played by lateral connections in implementing object-based attention and perceptual grouping (Mehrani & Tsotsos, 2023), which have also been shown to change dynamically in the retinotopic maps of the ventral pathway (Ramalingam, McManus, Li, & Gilbert, 2013; Roelfsema, 2023).

### Behavioral experiment

**Figure 2:**
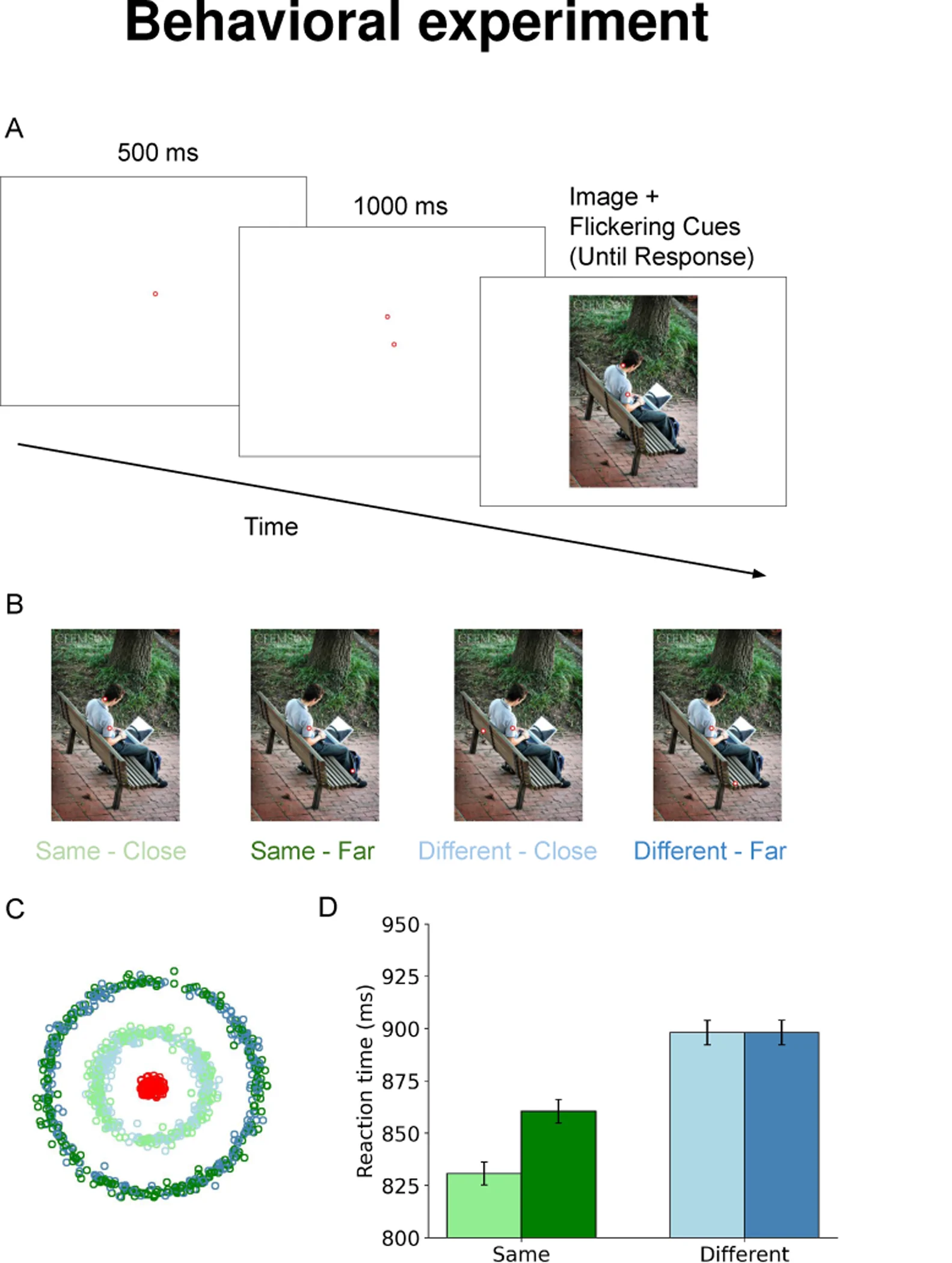
**A)** Behavioral procedure. Subjects maintain fixation on the center dot during the trial. **B)** Sample trial from all four conditions. **C)** Placement of dots across conditions across all trials. **D)** Mean reaction time by condition with SEM.

We use a ”two-dot” paradigm (Fig. 2) to directly probe how humans group and segment the regions of natural images into objects. In this paradigm, two dots are placed on an image and subjects are asked to indicate whether they are on the same object or two different objects with a button press (Fig. 2A). One of these dots is always at the center location, and the other is at a peripheral location. The reaction time (RT) of this button press is our primary measure. Previous works using this paradigm have been limited in scale or have focused on simpler stimuli (Vecera, 2000; Kim, Linsley, Thakkar, & Serre, 2019; Korjoukov et al., 2012). We selected images from the Microsoft COCO (Common Objects in Context) dataset, which has images of complex everyday scenes depicting common objects in their natural context (Lin et al., 2014). The images also come with object-level segmentations, which we used to generate four versions of each display, two with both dots on the same object (same condition) and the other two with dots on different objects (different condition), see Fig. 2B. Within the same and different conditions, the peripheral dots on each trial were placed either close to or far from the center dot (3 and 6 degrees of visual angle, respectively, equally divided) (Fig. 2C). We generated 1024 unique experimental trials and have collected behavioral data from 42 subjects with each performing the task for 256 trials. Fig. 2D shows the RT data, subjects were faster to respond when the two dots were on the same object. This same object advantage interacted with dot distance, where we observed the fastest RTs in the close-separation same-object condition. This behavioral pattern is consistent with the hypothesis that attention spreads from the center dot within the cued object, thereby reaching the closer dot faster than the farther dot. If the second dot is on a different object, dot separation would not be expected to play a large role on RTs (Roelfsema, 2023).

## Modeling Results

**Figure 3:**
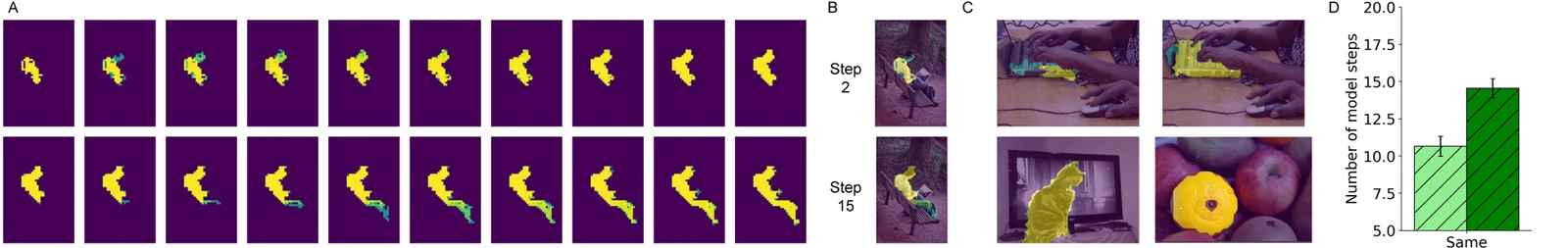
**A)** 20 steps of attention spread, starting from the center dot. **B)** Attention spread overlaid on the image for the steps that attention reached the second dot in the close (top) and far (bottom) conditions. **C)** Examples of attention spreading in objects. **D)** Mean number of steps for attention to reach the second dot in the close (light bar) and far (dark bar) conditions, with SEMs.

In transformers, each token is represented with three vectors: key, query, and, value. The affinity between tokens can be calculated using any of these feature representations (self-attention is the dot product of one token’s key with another token’s query). Following prior work that showed the key features to be the most object-centric (Wang et al., 2022), we calculate affinity by performing the dot product of each token’s key with all the other keys that we extracted from the last transformer layer of the DINO model with images as input (Caron et al., 2021) (Fig. 1 shows the affinity maps for a few patches). The model, like the subjects, starts every trial from the patch at the center dot location. Then, from this starting location, the model selects all the tokens with strong affinity above a threshold, causing attention to spread to a bigger segment around the center dot (Fig. 3A top-left). At each new step, the model identifies all the patches that have a strong affinity to the already attended segment by taking an average across all affinity maps from all the tokens in the growing segment. Fig. 3A shows the iterative spread of attention in an object over 20 steps. As the segment grows, the attention spread becomes more conservative due to the constraint placed on the segment growth to be a single connected region and that all the patches in the segment vote where to spread next. To counter this conservative spread, the model reduces the threshold at each time step. The number of steps that it takes for attention to reach the peripheral dot becomes the model’s prediction of the RT for that trial. Fig. 3B shows the attention spread overlaid on the original image at steps 2 and 15, where it reached the close (top) and far (bottom) peripheral dots (see Fig. 3C for more examples). Focusing analyses on only the same trials, we plot in Fig. 3D the average number of steps that the model took for attention to reach the close and far dots. The model predicts the same effect of distance on RT as we saw in humans. While this shows that the model on average predicts the spreading of object-based attention in humans, future work will aim to evaluate the model on predicting human behavior on individual trials, including on the different condition.

## Discussion

Several important insights follow from our work. First, just as Convolutional and Recurrent networks trained on object classification have been shown to predict behavior and neural activity during visual recognition (Cadieu et al., 2014; Kriegeskorte, 2015), we show that transformers can provide plausible mechanisms of visual functions beyond core object recognition, such as attention modulated perceptual grouping of features into objects. Second, the self-supervised training objective can provide a biologically plausible feature learning mechanism for how the primate visual system learns to group and perceive objects, one that does not require a huge number of labeled samples. Lastly, this work contributes to computer vision by showing how object-based attention, a core element of human cognition, can be integrated into an AI model.
